# Time management training and its effect on head nurses’ work-family conflict

**DOI:** 10.1186/s12912-025-03691-9

**Published:** 2025-11-21

**Authors:** Mansoura Aid Abed El Salam, Samah Faisal Fakhry, Samah Mohamed Elsayed

**Affiliations:** Nursing Administration, Faculty of Nursing, Ain Shams University, Cairo, Egypt

**Keywords:** Time management, Work-family conflict, Head nurses, Nursing leadership, Quasi-experimental design, Nursing administration, Training intervention

## Abstract

**Background:**

Head nurses frequently experience work-family conflict due to demanding managerial responsibilities and role overload. Time management is a critical skill that can enhance professional efficiency while promoting better balance between work and personal life. However, limited empirical evidence exists on the effectiveness of time management training in reducing work-family conflict among nursing leaders.

**Aim:**

To assess the effect of a structured time management training program on head nurses’ knowledge, time management practices, and work-family conflict levels.

**Methods:**

A quasi-experimental one-group pretest–posttest design was used. The study was conducted at a maternity hospital affiliated with Ain Shams University in Cairo, Egypt. A purposive sample of 40 head nurses participated. Data were collected using three tools: a time management knowledge questionnaire, an observational checklist for time management practices, and the Work-Family Conflict Scale. The intervention consisted of eight interactive training sessions over four weeks. Data were collected at three time points: pre-intervention, immediately post-intervention, and at a three-month follow-up. Descriptive statistics, chi-square tests, repeated-measures ANOVA, and multiple regression analysis were used.

**Results:**

Post-intervention assessments showed significant improvements in knowledge (from 42.6 to 97.6%) and observed time management practices (e.g., delegation improved from 12.3 to 98.9%). There was also a significant reduction in all domains of work-family conflict, with the proportion reporting high conflict dropping from 72.3 to 14.7%. Regression analysis identified prior training and years of experience in the current role as significant predictors of improved outcomes (*p* < 0.05).

**Conclusion:**

Time management training is an effective intervention for enhancing head nurses’ performance and reducing their perceived work-family conflict. Incorporating such training into leadership development programs may support nurse well-being and organizational efficiency.

**Clinical trial:**

Not applicable.

## Introduction

The healthcare sector has undergone transformative changes in recent decades, resulting in increasing demands on nursing leaders to balance complex administrative roles with their professional and personal lives [1]. Among these leaders, head nurses bear a critical responsibility for managing nursing units, ensuring patient care quality, overseeing staff performance, and aligning nursing operations with institutional goals. These multifaceted demands often culminate in considerable time pressure, occupational stress, and difficulties balancing work and family roles [2]. As such, effective time management is not merely an administrative skill for head nurses—it is a vital competency linked to organizational efficiency, personal well-being, and sustainable workforce engagement [3].

Time management refers to a systematic process of planning and controlling how much time is spent on specific activities to enhance effectiveness, efficiency, and productivity [4]. In the nursing context, effective time management can mitigate professional burnout, reduce occupational stress, and enhance job satisfaction, particularly for nurses in leadership positions [2]. Head nurses, in particular, are frequently confronted with administrative overload, constant interruptions, conflicting demands, and staffing challenges. These stressors, if not properly managed, can undermine both professional performance and personal well-being [5]. Previous research has demonstrated that time mismanagement can lead to suboptimal care delivery, increased turnover, and diminished quality of life among healthcare providers [6].

Nursing leadership roles, especially those of head nurses, are increasingly shaped by the dual pressures of clinical supervision and administrative obligations. The head nurse is expected to coordinate staff schedules, resolve interpersonal conflicts, oversee resource allocation, and simultaneously ensure compliance with healthcare standards [7]. These competing responsibilities often require the nurse leader to engage in multitasking and decision-making under time constraints. Without adequate training in time management strategies, head nurses may struggle to meet expectations, leading to decreased job satisfaction and impaired work-life balance [8]. Moreover, the increasing complexity of healthcare systems, driven by technological advancements and organizational restructuring, has amplified the need for structured time management training in nursing leadership development programs [9].

Parallel to the growing demands within the workplace, head nurses also face escalating pressures within their family and personal lives. The intersection between professional responsibilities and family obligations is increasingly being recognized as a source of work-family conflict (WFC)—a bidirectional phenomenon where work demands interfere with family roles and vice versa [10]. WFC is associated with psychological strain, emotional exhaustion, diminished job performance, and poor family relationships [11]. In particular, women in nursing leadership positions often experience heightened WFC due to gendered expectations regarding caregiving and household responsibilities [12]. The prevalence of WFC among head nurses not only jeopardizes individual well-being but may also impair organizational functioning through absenteeism, turnover, and reduced morale [13].

Work-family conflict has been conceptualized across three dimensions: time-based conflict, strain-based conflict, and behavior-based conflict. Time-based WFC occurs when the time committed to professional tasks reduces the time available for family activities. Strain-based WFC refers to emotional and psychological burdens at work spilling over into the family domain. Behavior-based WFC involves the incompatibility between behavioral expectations in the workplace and those required in the family environment [14]. These dimensions are particularly relevant in nursing, where shift work, unpredictable schedules, and emotional labor are pervasive.

Effective time management may serve as a mitigating strategy to reduce WFC among nursing leaders. By enhancing the ability to prioritize tasks, delegate responsibilities, and avoid time wasters, head nurses can gain more control over their schedules and reduce the conflict between work and family domains [4]. Time management training programs that focus on planning, goal setting, prioritization, and handling interruptions have been shown to improve professional efficacy and reduce emotional strain in various occupational groups, including healthcare professionals [15, 16]. However, empirical research investigating the direct impact of time management training on WFC among head nurses remains limited, particularly in the context of developing healthcare systems.

In Egypt and similar Middle Eastern settings, head nurses frequently operate in under-resourced hospitals with high patient loads and insufficient staffing. The compounded burden of professional and familial duties can place these leaders at elevated risk for time-related stress and WFC [17]. Yet, despite their pivotal role in ensuring the operational success of healthcare facilities, there is a notable lack of structured leadership development interventions targeted at improving head nurses’ time management capabilities [18]. Given this gap, it is imperative to explore whether targeted time management training can equip head nurses with the skills necessary to manage their responsibilities more effectively and, in turn, alleviate their experience of work-family conflict [19].

This study is grounded in the premise that enhancing time management knowledge and skills among head nurses will lead to improved practice behaviors and a measurable reduction in perceived work-family conflict. It seeks to contribute to the growing body of literature emphasizing the importance of administrative training for nursing leaders, while also addressing the psychosocial dimensions of occupational health.

### Aim of the study

The present study aims to evaluate the effectiveness of a structured time management training program on the time management knowledge, practices, and work-family conflict levels among head nurses working at a maternity hospital affiliated with Ain Shams University.

### Research questions

1. What is the effect of the time management training program on head nurses’ knowledge and practice regarding time management?
2. To what extent does the time management training program influence the levels of work-family conflict among head nurses?
3. Are there significant predictors (e.g., years of experience, prior training) associated with changes in time management practices and work-family conflict outcomes post-intervention?

## Materials and methods

### Study design

A quasi-experimental one-group pretest–posttest design was adopted to evaluate the effectiveness of a structured time management training program on head nurses’ knowledge, practices, and work-family conflict levels. This design was chosen for its suitability in preliminary interventional studies where randomization is not feasible due to organizational or ethical constraints. By assessing participants at three time points—pre-intervention, immediately post-intervention, and three months post-intervention—the study aimed to capture both immediate and sustained changes attributable to the training. While the absence of a control group limits causal inference, the design provides valuable insight into within-subject change and supports hypothesis generation for future controlled studies.

### Study setting

The study was conducted at the Maternity Hospital affiliated with Ain Shams University Hospitals in Cairo, Egypt. This hospital is a tertiary care facility and academic teaching institution, which provides comprehensive maternity and neonatal services to a large catchment population. It comprises several nursing departments including obstetrics, gynecology, neonatal intensive care, and operating theaters, all managed by senior head nurses responsible for administrative and supervisory tasks.

### Sample and sampling

The target population comprised all head nurses employed at the Maternity Hospital affiliated with Ain Shams University Hospitals in Cairo, Egypt. At the time of the study, a total of 40 head nurses were working across the hospital’s clinical and administrative departments, including obstetrics, gynecology, neonatal intensive care, and surgical units. All 40 head nurses met the inclusion criteria and were invited to participate, making the sample representative of the hospital’s entire head nurse population. Their responsibilities encompass supervising nursing staff, ensuring quality patient care, coordinating workflow, and managing resources. Given the centrality of their position in healthcare delivery and their exposure to dual-role stressors (work and family), head nurses were deemed the most appropriate group for evaluating the impact of a time management training intervention on work-family conflict.

A purposive (non-probability) sampling technique was employed to select participants based on specific inclusion and exclusion criteria. This method was chosen to ensure that the selected sample would possess the relevant characteristics and experiences necessary to address the research objectives effectively. The inclusion criteria were: (1) currently holding the position of head nurse at the selected maternity hospital; (2) having a minimum of one year of continuous experience in a leadership or supervisory role; (3) willingness to voluntarily participate in the study; and (4) availability during the study period for the training sessions and follow-up assessments. The exclusion criteria included head nurses who were (1) on extended leave during the study period; (2) currently participating in other leadership or time management development programs; or (3) having undergone formal time management training within the last six months, which might confound the study outcomes.

Using this criteria-based approach, a total of 40 head nurses were recruited and consented to participate. This sample size was guided by practical feasibility, accessibility of the study population, and prior literature employing similar designs. To ensure the adequacy of the sample size for statistical analysis, a power analysis was conducted using G*Power 3.1 software. Assuming a moderate effect size (Cohen’s d = 0.5), an alpha level of 0.05, and a power of 0.80, the minimum required sample size for detecting statistically significant differences across three time points (pre-intervention, post-intervention, and follow-up) was calculated to be 34 participants. Therefore, enrolling 40 head nurses allowed for possible attrition while maintaining sufficient statistical power.

All participants were informed about the study purpose, procedures, and ethical considerations. They were assured that their participation would be entirely voluntary, and they could withdraw from the study at any point without consequence. Each participant provided written informed consent prior to data collection. None of the invited head nurses declined participation, and full retention was achieved across the intervention and follow-up phases, thus ensuring data completeness and consistency.

### Data collection tools

Data were collected using three structured tools designed to assess head nurses’ knowledge and practices regarding time management as well as their experience of work-family conflict.

#### Time management knowledge questionnaire

This questionnaire was developed by the researchers based on a comprehensive literature review [20] and aimed to assess participants’ theoretical understanding of time management concepts. It consisted of two parts. Part I captured demographic and job-related characteristics such as age, gender, years of experience, educational background, and previous training in time management. Part II assessed knowledge across 31 multiple-choice items divided into thematic areas: definition and importance of time management, benefits, key principles, time wasters, delegation techniques, and time management strategies. Each correct answer was awarded one point, while incorrect or missing responses received zero. A total knowledge score was calculated and converted into a percentage. Scores ≥ 60% were categorized as “satisfactory,” while scores < 60% were considered “unsatisfactory.” The tool’s internal consistency was confirmed with a Cronbach’s alpha of 0.745, indicating acceptable reliability. The questionnaire was translated into Arabic using forward–backward translation methods by bilingual nursing educators and linguists to ensure semantic and conceptual equivalence. The Arabic version was reviewed by a panel of five experts in nursing administration and education for content validity, achieving a Content Validity Index (CVI) of 0.91.

#### Time management practice observational checklist

This checklist, adapted from Kamal (2019), assessed observable time management behaviors in the clinical setting. To ensure the reliability of observational data, two trained nursing faculty members served as observers. Prior to data collection, they underwent a calibration session involving pilot observations and consensus scoring to standardize interpretation [21]. The tool aimed to objectively assess time management behaviors exhibited by head nurses in the clinical setting. It consisted of 10 observable domains including planning, setting goals, setting priorities, delegation, workplace organization, handling telephone interruptions, managing meetings, responding to unexpected visits, time control, and avoiding procrastination. Each item was scored as “done” (1) or “not done” (0), with total scores ranging from 0 to 10. Higher scores indicated better time management practice. The tool demonstrated excellent internal consistency with a Cronbach’s alpha of 0.804. Translation into Arabic followed the same rigorous protocol described above, with the validated Arabic version reviewed by an expert panel for cultural and professional appropriateness, yielding a CVI of 0.94. Inter-rater reliability was evaluated using Cohen’s kappa, which yielded a coefficient of 0.81, indicating strong agreement. Observers were assigned to participants randomly and rotated to minimize potential bias.

#### Work-family conflict scale

This instrument was originally and later adapted for nursing populations by Ebrahimi et al. [22]. The scale was designed to measure the extent of conflict between professional and familial roles. It consisted of 18 items distributed across six subscales: time-based work interference with family (WIF), time-based family interference with work (FIW), strain-based WIF, strain-based FIW, behavior-based WIF, and behavior-based FIW. Each subscale contained three items rated on a 5-point Likert scale ranging from 1 (strongly disagree) to 5 (strongly agree). Subscale scores were summed, and higher scores indicated greater levels of work-family conflict. The adapted Arabic version of the scale underwent standard translation and validation procedures. Reliability analysis produced a Cronbach’s alpha of 0.887 for the overall scale. Content validity of the translated version was confirmed by nursing faculty with expertise in psychology, administration, and instrument development.

### Data collection procedure

The study was conducted from January 2023 to November 2024. Following administrative approval, participants were informed about the study’s purpose and invited to provide written consent. Data collection occurred in four phases:

1. **Assessment Phase (Pre-intervention)**: Participants completed the knowledge questionnaire and WFC scale, and the observational checklist was applied in their units by trained observers.
2. **Planning Phase**: Based on assessment findings and literature review, the researchers developed an evidence-based training curriculum comprising eight sessions (2 h each) delivered over four weeks.
3. **Implementation Phase**: The training was delivered face-to-face in small groups (5–8 participants per session) using interactive lectures, case discussions, role play, and group exercises. Educational materials were provided in Arabic to enhance accessibility.
4. **Evaluation Phase**: The same tools were re-administered immediately post-intervention and three months later during follow-up. The observers remained blinded to the study objectives to reduce bias.

Observational assessments were conducted in the participants’ clinical units using a standardized protocol. To reduce observer bias, the individuals conducting observations were not involved in the training sessions and were blinded to the study’s hypotheses and participant performance status (i.e., pre/post/follow-up phase). Each head nurse was observed by two independent raters over the course of a standard work shift, and discrepancies were resolved through discussion and consensus.

### Intervention content and delivery

The time management training program consisted of eight structured sessions, each lasting approximately two hours, delivered over a four-week period (two sessions per week). The program was developed based on a comprehensive literature review and aligned with best practices in leadership and time management training. It was tailored to the managerial and administrative responsibilities of head nurses in a maternity hospital setting.

Each session focused on a specific learning objective:

1. **Introduction to Time Management**: Understanding definitions, benefits, and the impact of poor time use.
2. **Setting SMART Goals**: Techniques for setting measurable, actionable short- and long-term goals.
3. **Prioritization and Planning**: Use of tools like Eisenhower Matrix and daily scheduling templates.
4. **Identifying and Controlling Time Wasters**: Recognizing internal and external interruptions and managing them effectively.
5. **Delegation and Decision-Making**: Enhancing productivity through task delegation and boundary-setting.
6. **Managing Meetings and Communication**: Strategies for efficient communication and reducing time lost in meetings.
7. **Stress and Procrastination Management**: Time-related stress awareness and behavioral techniques to overcome procrastination.
8. **Work-Life Integration Strategies**: Balancing administrative duties with personal and family responsibilities.

The sessions were delivered by two senior nurse educators with doctoral degrees in nursing administration and more than 10 years of experience in leadership development and adult learning. The instructional strategies included interactive lectures, case-based discussions, role play, simulation, and group exercises. Educational handouts, worksheets, and time audit tools were provided in Arabic to maximize comprehension and applicability. Small-group sessions (5–8 participants) facilitated engagement and peer learning.

### Data analysis

Data were analyzed using IBM SPSS Statistics version 26. Descriptive statistics (frequency, percentage, mean, and standard deviation) summarized participants’ characteristics and outcome measures. For categorical variables representing observed time management behaviors across three time points (pre, post, and follow-up), Cochran’s Q test was used to account for within-subject repeated measures of dichotomous variables. Where Cochran’s Q was significant, McNemar’s post-hoc pairwise comparisons with Bonferroni correction were applied.

For continuous variables (e.g., total Work-Family Conflict scores), repeated-measures ANOVA was conducted. Assumption checks included Mauchly’s test of sphericity; where the assumption was violated, Greenhouse-Geisser corrections were applied. Effect sizes were calculated and reported as partial eta squared (η²) to quantify the magnitude of observed changes. A multiple linear regression model was used to identify predictors of post-intervention WFC outcomes. Statistical significance was set at *p* < 0.05.

### Ethical considerations

This study was conducted in compliance with the ethical principles outlined in the Declaration of Helsinki. Ethical approval was obtained from the Institutional Review Board (IRB) of the Faculty of Nursing, Ain Shams University. Administrative approval was also secured from the Director of the Maternity Hospital. All participants were informed about the voluntary nature of their participation, the confidentiality of their responses, and their right to withdraw from the study at any time without penalty. Written informed consent was obtained prior to data collection. Participants’ data were anonymized and stored securely with access limited to the research team.

## Results

### Demographic and job-related characteristics of participants

Table 1 summarizes the demographic and professional characteristics of the study participants. The sample predominantly comprised female head nurses (90.2%), with the majority aged between 40.2 and 49.8 years (42.7%). Nearly two-thirds (67.3%) had more than 15.3 years of nursing experience, and approximately half (52.8%) held a Bachelor of Nursing degree. Only 32.3% had previously participated in formal time management training.

**Table 1:** Demographic and professional characteristics of study participants (*N* = 40)

Variables	Categories	*n* (%)
Age	30–39 years	7 (17.5%)
	40–49 years	17 (42.5%)
	≥ 50 years	16 (40.0%)
Gender	Female	37 (90.2)
	Male	4 (9.8)
Education	Bachelor	22 (52.8)
	Diploma	18 (47.2)
Nursing Experience	< 10.1 yrs	3 (7.7)
	10.2–15.2 yrs	10 (25.0)
	> 15.3 yrs	27 (67.3)
Prior Training	Yes	13 (32.3)
	No	27 (67.7)

### Knowledge about time management (pre, post, follow-up)

Table 2 presents the participants’ knowledge regarding different domains of time management across three assessment phases: pre-intervention, immediate post-intervention, and follow-up after three months. Initially, participants demonstrated moderate knowledge (67.3%) in the conceptual understanding of time management, whereas knowledge was lower regarding the identification (34.8%) and management (32.8%) of time wasters, as well as the implementation of specific time management strategies (42.6%). Following the intervention, significant improvements occurred in all knowledge domains, with post-intervention scores surpassing 95% across domains. At the three-month follow-up assessment, these high knowledge levels were largely sustained, indicating enduring effects of the training. Statistical analysis confirmed these observed differences as highly significant (*p* < 0.001) for comparisons between pre-intervention and both post-intervention and follow-up assessments, underscoring the intervention’s impact on enhancing and maintaining time management knowledge among the participants.

**Table 2:** Knowledge about time management across phases (*N* = 40)

Domain	Pre (%)	Post (%)	Follow-Up (%)	Cochran’s Q	*p*-value
Concept of Time Management	67.5%	98.8%	97.5%	32.54	< 0.001
Identification of Time Wasters	35.0%	96.8%	92.0%	35.67	< 0.001
Management of Time Wasters	32.5%	95.0%	94.0%	34.21	< 0.001
Time Management Strategies	42.5%	97.5%	91.8%	31.89	< 0.001

Note: Cochran’s Q test was used to assess within-subject differences across three repeated measures. All differences were statistically significant (*p* < 0.001)

### Time management practices

The data presented in Table 3 highlight notable changes in head nurses’ observed time management practices across three assessment points: pre-intervention, post-intervention, and follow-up. Prior to the implementation of the training program, the percentages of nurses exhibiting effective behaviors in domains such as planning (34.7%), setting goals (37.6%), and setting priorities (29.9%) were relatively modest. Practices such as delegation and avoiding procrastination were even less frequently observed, reported at only 12.3% and 12.2%, respectively. Following the intervention, a marked increase was noted in all domains, with post-intervention scores exceeding 95% across the board. Specifically, 98.1% of participants demonstrated effective planning behaviors, 95.2% set goals, and nearly 99% practiced delegation. Notably, avoidance of procrastination reached 100%, indicating full adherence to this practice among participants immediately after training. These improvements were sustained at the three-month follow-up, with only slight variations. The chi-square analysis revealed statistically significant differences between pre- and post-intervention as well as between pre- and follow-up measurements (*p* < 0.001 for all domains), suggesting that the observed enhancements were both substantial and sustained over time. The data collectively suggest a strong association between the training program and the development of practical time management skills among head nurses.

**Table 3:** Observed time management practices (*N* = 40)

Practice Domain	Pre (%)	Post (%)	Follow-Up (%)	Cochran’s Q	*p*-value
Planning	35.0%	98.0%	96.3%	38.26	< 0.001
Setting Goals	37.5%	95.0%	97.5%	36.72	< 0.001
Setting Priorities	30.0%	97.5%	98.0%	37.81	< 0.001
Delegation	12.5%	98.8%	97.5%	35.34	< 0.001
Avoiding Procrastination	12.5%	100.0%	100.0%	36.97	< 0.001

Note: Cochran’s Q test showed statistically significant differences in all practice domains across the three time points. All p-values were < 0.001

### Work-family conflict (time-based, strain-based, behavior-based)

Table 4 presents the distribution of head nurses experiencing high levels of work-family conflict (WFC) across three distinct domains—time-based, strain-based, and behavior-based—at three time points: pre-intervention, post-intervention, and follow-up. The data reveal a consistent pattern of reduction in high-level WFC across all domains following the implementation of the time management training program. At baseline, a substantial proportion of participants reported high levels of conflict: 72.3% for time-based WFC, 74.7% for strain-based WFC, and 69.8% for behavior-based WFC. These figures reflect a notable degree of interference between professional obligations and family responsibilities prior to the intervention. Following the training, the percentages of participants reporting high WFC dropped markedly in all domains—to 14.7% for time-based, 13.9% for strain-based, and 14.8% for behavior-based WFC. At the three-month follow-up, slight increases were observed; however, the percentages remained significantly lower than pre-intervention levels, with 19.8%, 18.9%, and 20.3% respectively. The Chi-square test results indicated statistically significant differences between pre- and post-intervention, as well as between pre- and follow-up assessments in all domains (*p* < 0.001).

**Table 4:** Levels of Work-Family conflict by domain (*N* = 40)

Conflict Domain	Level	Pre (%)	Post (%)	Follow-Up (%)	χ² (Pre-Post)	*p*-value	χ² (Pre-Follow-Up)	*p*-value
Time-Based WIF	High	72.3	14.7	19.8	19.542	< 0.001	18.923	< 0.001
Strain-Based WIF	High	74.7	13.9	18.9	20.324	< 0.001	17.896	< 0.001
Behavior-Based WIF	High	69.8	14.8	20.3	21.472	< 0.001	19.045	< 0.001

### Overall work-family conflict scores

Table 5 presents the mean total scores of work-family conflict across the three assessment phases: pre-intervention, post-intervention, and follow-up. Prior to the implementation of the time management training program, participants reported a relatively high level of work-family conflict, with a mean score of 67.2 (± 9.6). Following the intervention, there was a marked reduction in the mean score to 23.4 (± 5.3), suggesting an improvement in participants’ ability to balance professional and familial responsibilities. At the three-month follow-up, the mean score slightly increased to 27.1 (± 6.1), yet remained substantially lower than the baseline level. Statistical analysis using repeated-measures ANOVA revealed a highly significant difference across the three time points (F = 45.236, *p* < 0.001), indicating a sustained reduction in perceived work-family conflict. Repeated-measures ANOVA revealed a statistically significant reduction in total Work-Family Conflict scores across the three time points (F(2, 78) = 45.236, *p* < 0.001, partial η² = 0.61), indicating a large effect. Mauchly’s test confirmed that the sphericity assumption was met (*p* = 0.13), and thus no correction was applied.

**Table 5:** Total Work-Family conflict scores (*N* = 40)

Assessment Phase	Mean ± SD	F-value	*p*-value
Pre-Intervention	67.2 ± 9.6	45.236	< 0.001
Post-Intervention	23.4 ± 5.3		
Follow-Up	27.1 ± 6.1		

### Regression analysis of predictors for work-family conflict

Table 6 presents the regression analysis identifying factors associated with changes in work-family conflict scores among head nurses. The model explains 39.8% of the variance (R² = 0.398) and is statistically significant (F = 4.013, *p* < 0.05). Two variables were significant predictors: years of experience in the current job (β = 2.489, *p* = 0.013) and prior time management training (β = 2.113, *p* = 0.021). This indicates that longer tenure and previous exposure to time management strategies are linked to more favorable outcomes. Age showed a positive association (β = 1.047) but was not statistically significant (*p* = 0.122). These findings suggest that both experiential and developmental factors influence how head nurses respond to interventions aimed at reducing work-family conflict.

**Table 6:** Predictors of Work-Family conflict (*N* = 40)

Predictor Variables	β	SE	t-value	*p*-value
Age (years)	1.047	0.378	2.768	0.122
Years of Experience in Current Job	2.489	1.364	3.168	0.013
Prior Time Management Training	2.113	1.162	2.672	0.021

Model: F = 4.013, R²=0.398, *p* < 0.05

## Discussion

This study aimed to evaluate the effectiveness of a structured time management training program on improving head nurses’ time management knowledge and practices and reducing their perceived work-family conflict. The findings indicate significant improvements in all targeted outcomes following the intervention, with effects sustained at the three-month follow-up.

The results demonstrated a marked enhancement in participants’ knowledge related to key domains of time management, including its concept, identification and control of time wasters, and application of strategic approaches. This aligns with findings from prior studies emphasizing that educational interventions can substantially improve cognitive understanding of time use among nursing leaders [23, 24]. The sustained improvement observed at follow-up suggests not only successful knowledge acquisition but also retention, which may be attributed to the practical relevance of the content, the interactive nature of the training, and the participants’ leadership responsibilities that likely reinforced continued application [25, 26].

In addition to knowledge gains, the observational data revealed substantial improvements in the actual time management behaviors of head nurses, particularly in critical areas such as planning, goal setting, delegation, and procrastination avoidance [27]. These findings are consistent with earlier research demonstrating the positive impact of skill-based training programs on managerial efficiency and nursing performance [28]. The improvements in delegation and priority setting are particularly noteworthy, as these skills are essential for managing workload and minimizing disruptions in high-acuity clinical environments [29, 30].

One of the most salient outcomes of the study was the significant reduction in work-family conflict across all measured domains—time-based, strain-based, and behavior-based conflict [31]. This reduction was most prominent immediately post-intervention and remained statistically significant at follow-up. These results echo findings from Khatatbeh (2022) and Wise (2022), who reported that improved time management skills were associated with decreased emotional exhaustion and improved role balance among nurses. The ability to manage work demands more efficiently appears to translate directly into reduced spillover into personal life domains [32].

The bidirectional nature of work-family conflict underscores the relevance of addressing both professional competencies and psychosocial wellbeing [33]. As shown in this study, enhancing a nurse leader’s time control may buffer against stressors that lead to conflict between work and family responsibilities. The consistent decrease in high WFC scores supports the theoretical framework that improved self-regulation and planning can reduce perceived role strain.

The regression analysis further contributes to understanding the contextual factors influencing intervention effectiveness [34–39]. Specifically, years of experience in the current job and prior time management training emerged as significant predictors of reduced work-family conflict. This suggests that experienced head nurses with foundational time management exposure may be more capable of integrating advanced techniques into daily practice. These findings align with those of Aldhafeeri et al. (2025), who noted that cumulative professional development enhances coping strategies and organizational adaptation [13].

Interestingly, while age showed a positive association with WFC reduction, it did not reach statistical significance. This may reflect variability in personal circumstances or generational differences in coping mechanisms and domestic responsibilities [40]. It may also suggest that professional experience, rather than age alone, better predicts responsiveness to leadership development interventions [41].

The findings have several practical implications. First, they support the integration of time management training into continuing professional development programs for nursing leaders. Structured training can enhance not only individual effectiveness but also contribute to a more balanced work-life interface, which is critical for retention and job satisfaction. Second, the results highlight the importance of tailoring interventions based on experience level and prior training history to maximize impact.

### Implications for nursing practice and policy

The findings of this study have important implications for nursing leadership development, administrative practice, and workforce sustainability. The statistically significant improvements in time management knowledge, observed leadership behaviors, and reductions in work-family conflict support the integration of structured time management training into professional development curricula for nurse leaders. Such training not only enhances individual administrative efficiency but also contributes to improved role balance and psychological well-being—key factors in nurse retention and performance.

From a policy standpoint, healthcare institutions should consider embedding evidence-based time management modules into mandatory training programs for head nurses and mid-level managers. Institutional support structures—such as protected time for training, delegation frameworks, and workload management protocols—may amplify the impact of individual skill development. Furthermore, the sustained benefits observed at three-month follow-up highlight the potential for long-term leadership capacity building when training is reinforced through ongoing support or refresher modules.

Given the association between prior training, years of experience, and improved outcomes, a tiered or competency-based approach to leadership training may help tailor interventions to individual readiness levels. These strategies are particularly relevant in resource-constrained healthcare systems where nurse managers are expected to perform under significant time pressure and staffing challenges. Ultimately, time management training should be viewed not only as a technical intervention but as a strategic investment in nurse leader resilience, job satisfaction, and organizational health.

### Limitations

While this study provides valuable insights into the effects of time management training on head nurses’ work-family conflict and leadership practices, several limitations should be acknowledged. First, the quasi-experimental one-group pretest–posttest design limits the ability to draw definitive causal inferences, as the absence of a control or comparison group introduces potential threats to internal validity, including history, maturation, and testing effects. Future studies should consider using randomized controlled trials or matched-group designs to strengthen causal attribution.

Second, the study relied on self-reported measures to assess knowledge and perceived work-family conflict. Although validated instruments were used, self-reporting is inherently subject to biases such as social desirability and recall error. While we included an observational checklist to capture behavioral data and minimize bias, future research would benefit from triangulating findings with objective indicators such as productivity records, attendance logs, or supervisor evaluations.

Third, although all eligible head nurses at the study site were included, the sample was drawn from a single maternity hospital in Egypt, and the majority of participants were experienced female nurses. This homogeneity limits the generalizability of the findings to other healthcare contexts, such as non-maternity settings, mixed-gender leadership populations, or hospitals in other cultural or geographic regions. Multisite studies with more diverse samples are recommended to enhance external validity.

Fourth, the follow-up period was limited to three months. While sustained effects were observed at follow-up, a longer evaluation window (e.g., six to twelve months) would allow for better assessment of the durability of knowledge retention, practice change, and conflict reduction over time.

Fifth, although regression analysis was used to identify predictors of outcome improvement, potential moderating variables—such as institutional support structures, family demands, or organizational workload—were not explored. Future studies could benefit from examining how such contextual factors influence intervention effectiveness.

Finally, the study did not include a qualitative component to capture participants’ lived experiences, perceived barriers, or facilitators to behavior change. Incorporating interviews or focus groups in future research would enrich understanding of the mechanisms underlying time management behavior change and its impact on work-family dynamics.

## Conclusion

This study provides empirical evidence that structured time management training can significantly enhance head nurses’ knowledge and practice behaviors while reducing their perceived work-family conflict. The intervention led to sustained improvements in time planning, goal setting, delegation, and avoidance of procrastination—core competencies essential for nursing leadership. Concurrently, there was a marked and statistically significant decline in time-, strain-, and behavior-based work-family conflict, with effects largely maintained at three months post-intervention.

Importantly, regression findings indicated that prior exposure to time management concepts and professional experience predicted better outcomes, underscoring the value of both foundational training and experiential learning in leadership development. These results suggest that time management training is not only effective in the short term but may also be scalable across diverse healthcare settings as part of leadership capacity-building efforts.

Nonetheless, given the study’s single-site design and absence of a control group, the findings should be interpreted with caution. Future studies should explore the long-term sustainability of these effects using randomized or matched-group designs, examine potential moderating influences such as institutional support and family structure, and incorporate qualitative insights to deepen understanding of behavioral change processes.

In summary, time management training represents a practical, evidence-informed strategy to strengthen head nurse performance and well-being—an essential step toward advancing nursing leadership and organizational resilience.

## Data Availability

The datasets generated and/or analyzed during the current study are not publicly available due to participant confidentiality and institutional policy but are available from the corresponding author on reasonable request.
